# The Aryl Hydrocarbon Receptor: A Target for Breast Cancer Therapy

**DOI:** 10.4236/jct.2013.47137

**Published:** 2013-09

**Authors:** Joann B. Powell, Gennifer D. Goode, Sakina E. Eltom

**Affiliations:** 1 Department of Biological Sciences & Center for Cancer Research and Therapeutic Development, Clark Atlanta University, Atlanta, USA; 2 Department of Biochemistry & Cancer Biology, Meharry Medical College, Nashville, USA

**Keywords:** Aryl Hydrocarbon Receptor, Therapeutic Targeting, Breast Cancer Progression, Chemosensitization

## Abstract

The aryl hydrocarbon receptor (AhR) is a ligand-activated transcription factor that regulates a battery of genes in response to exposure to a broad class of environmental poly aromatic hydrocarbons (PAH). AhR is historically characterized for its role in mediating the toxicity and adaptive responses to these chemicals, however mounting evidence has established a role for it in ligand-independent physiological processes and pathological conditions, including cancer. The AhR is overexpressed and constitutively activated in advanced breast cancer cases and was shown to drive the progression of breast cancer. In this article we will review the current state of knowledge on the possible role of AhR in breast cancer and how it will be exploited in targeting AhR for breast cancer therapy.

## 1. Introduction

Breast cancer is the second leading cause of cancer-related death in women in the US [1]. An estimated 40 percent of breast cancer patients relapse and develop metastatic disease and approximately 40,000 women die of breast cancer each year [2]; a mortality rate is largely attributed to systemic metastatic disease [3]. Despite the recently reported decline in death rates the complexity of breast cancer makes treatment of many breast cancer subtypes difficult [4,5]. Therefore, it is imperative to identify and characterize factors associated with breast cancer malignancy, which will have the potential to serve as novel molecular targets for breast cancer therapy. The AhR is one of these emerging factors that have the potential to be targeted for treating certain subtypes of metastatic breast cancer.

The AhR is a member of the basic-helix-loop-helix (bHLH)-Per-ARNT-Sim (PAS) family of transcription factors [6]. It is the only known ligand-activated member of the bHLH-PAS family [7]. It is well characterized for mediating the effects of a large class of polycyclic and poly halogenated aromatic hydrocarbons [8]. However, a growing body of literature suggests a role for AhR protein that diverges from its role as a sensor and adapter for exposure to environmental xenobiotics [9,10]. In particularly, the AhR proved to play a central role in driving the normal mammary gland development, and in an analogous fashion to drive the breast cancer progression. Although this dual double-edge role may make AhR a rather difficult target for breast cancer therapy, the focus will be on the unique aspects of its biology that is more specific to breast cancer invasion and metastasis.

## 2. Discussion

### 2.1. Role of AhR in Breast Development

The mammary gland is a complex tissue that undergoes many structural and functional changes during various life stages [11]. The AhR may play a role in breast development *in utero*, during pregnancy and is also reported to play a critical role in breast cancer development. AhR mRNA and protein is expressed as early as gestational day 10 – 16 in some embryonic mouse tissue [12]. AhR deficient mice exhibit altered development in multiple organs, including the mammary gland. Lack of AhR in the mammary gland results in a 50% decrease in terminal end buds [13]. *In utero* exposure to the prototypic AhR agonist, 2,3,7.8,-tetrachlorodibenzo-p-dioxin (TCDD), increases the number of terminal end buds and causes sustained defects in mammary gland development and functions in normal mice [14]. TCDD exposed mice also demonstrate reduced epithelial elongation and fewer alveolar buds. Evidence suggests that the alterations to mammary development are permanent in gestationally exposed animals. Mice exposed to TCDD *in utero* exhibit stunted progression of epithelium through the fat pad, fewer lateral branches and delayed lobule formation that persist past postnatal 68 [15]. However, TCDD exposed mammary glands retain the ability to differentiate in response to estrogen. TCDD exposed tissues express increased levels of estrogen receptor alpha and upon stimulation with estrogen induce mammary gland differentiation. The percentage of lobules I and II in TCDD exposed mammary glands increased significantly following exposure to 17β-estradiol [16]. Pregnant dams exposed to TCDD by gavage also demonstrated severe developmental defects including decreased mammary gland weight and branching. Analysis of hormone levels revealed a significant decrease in prolactin and estrogen on day 17 of pregnancy and at parturition [17]. These phenotypic changes mirror image those of the AhR null mice, underscoring the significance of the lack of the receptor or its activation with subsequent ligand-dependent depletion during those critical time of development.

AhR activation by TCDD during pregnancy has also been reported to delay DMBA-induced tumor formation in adult mice. TCDD exposure resulted in a 4-week delay in tumor formation. Overall tumor incidence was also lower in TCDD exposed group compared to the control group [18]. This is in contrast to alteration of mammary gland differentiation during *in utero* exposure, which is correlated with increased susceptibility to carcinogenesis. Prenatal TCDD treatment resulted in increased tumor incidence in rats [19]. Varying responses to TCDD exposure at different times during pregnancy have been reported [14]. Additional research is needed to determine if these diverse effects are a result of circulating estrogen levels or AhR protein levels.

Transcriptional pattern analysis revealed that AhR and AhR related genes are frequently deregulated in breast cancer. The majority of tumors tested revealed deregulation of AhR related genes [20]. Evaluation of AhR mRNA levels in rat mammary tissue and tumors indicates lower AhR expression in normal mammary epithelial cells in contrast to high AhR levels in DMBA-induced tumors [21]. Together, these findings suggest that AhR mediated mammary tumorigenesis may not require ligand-induced alteration of mammary gland structure and function.

### 2.2. AhR and Breast Cancer Progression

Elevated levels of AhR expression in human mammary tumors were reported from different laboratories including ours [22,23]. We reported dramatic elevated levels of AhR proteins in human breast carcinoma (HBC) cell lines from advanced malignancy (MDA-MB231, MDA-MB468, MDA-MB435s, MT2, NT, MCF7 breast cancer cell lines), while less levels were expressed in HBC derived from early stages of malignancy (T47D, MDA-MB-436 cell lines) and in immortalized and primary human mammary epithelial cells. The AhR was also constitutively activated in the advanced malignant cell lines [22]. Our observation on the breast cancer cell lines was later confirmed by others [23] who showed that infinite life-span cell lines had low levels of AhR mRNA compared to immortalized but non-malignant cell lines, which showed a 10-fold increase in AhR mRNA expression. Fully malignant cell lines had an 8-fold increase in AhR expression compared to the normal representative cell lines.

We further investigated the potential of AhR as a stage specific marker of breast cancer. We examined the expression of AhR by immunohistochemistry in tissue microarrays (TMA) containing 192 specimens of clinically defined three stages of invasive breast cancer: node negative, node-positive and metastatic carcinoma. Statistical analysis showed a highly significant correlation between the AhR expression and the carcinoma case type and the stage of invasive carcinoma (Eltom, *et al*., unpublished data). These findings provide compelling evidence for AhR as a new predictive clinical marker for metastatic breast cancer and a unique target for the design of novel selective inhibitors for therapeutic intervention of metastatic breast cancer.

**Figure 1:**
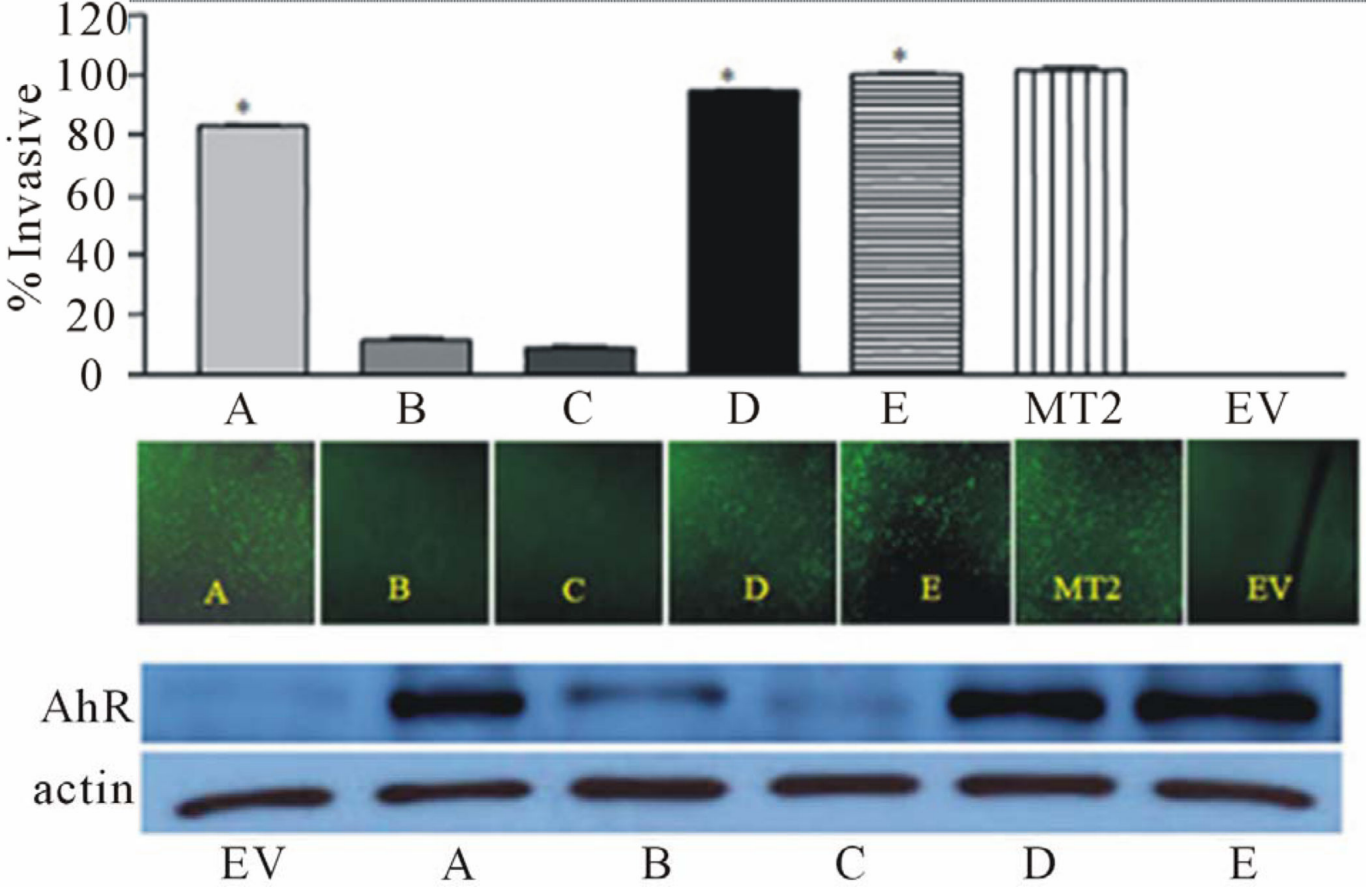
AhR is ectopically over expressed in clones (A–E) of an immortalized normal mammary epithelial cells (EV). Over expression of AhR induced invasion to a degree that correlated to the increase in AhR expression (from Brooks *et al*., 2011, with permission).

To delineate the role of AhR in the breast cancer progression, we employed the knock-in and knock-down approaches. First, we stably expressed high levels of AhR protein in an immortalized normal mammary epithelial cell line. Characterizing these transformed cells reveals that they exhibit several malignant properties. These cells underwent epithelial-mesenchymal-transition, had increased growth rates, abrogated cell cycle control and increased migration and invasive potential [24] (Figure 1). These studies revealed that AhR alone is required and sufficient to induce malignant transformation in mammary epithelial cells.

**Figure 2:**
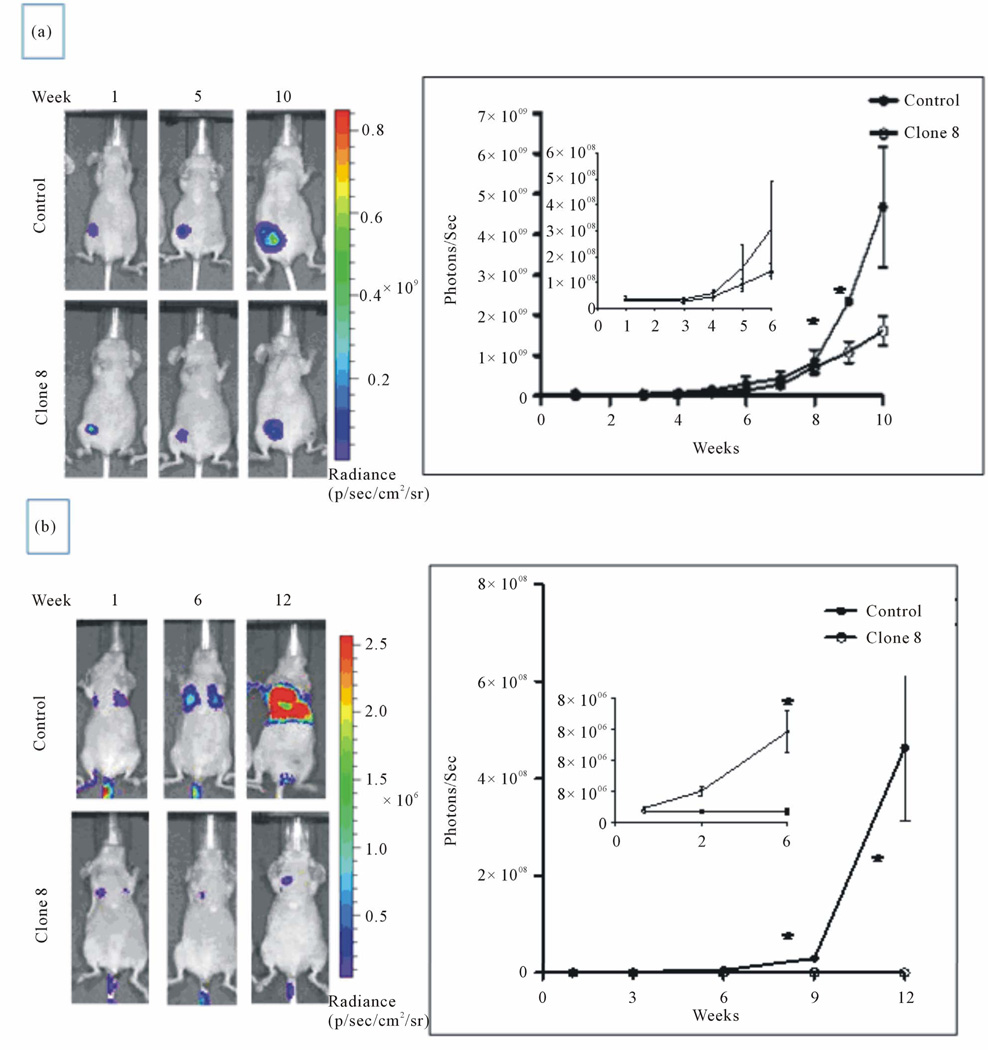
AhR knockdown reduces orthotopic growth of MDA-MB-231 cells in xenograft nude mice model (a) and reduces lung metastas is in nude mice experimental metastasis model (b) (from Goode *et al*., 2013, with permission).

We also showed that conversely, the stable knock down of inherently high levels of AhR in MDA-MB-231 human metastatic breast cancer cell line has resulted in attenuation of the tumorigenic properties *in vitro*, including proliferation, anchorage independent growth and migration while it enhanced their apoptosis. Testing the cells with depleted AhR for their ability to form tumors and metastasize in nude mouse xenograft model of breast cancer, we uncovered that lack of AhR has resulted in substantial reduction of the orthotopic xenograft tumor growth and experimental lung metastasis [25] (Figure 2), underscoring the critical role of AhR in driving both the tumor survival and metastasis.

### 2.3. Constitutive AhR Signaling

**Figure 3:**
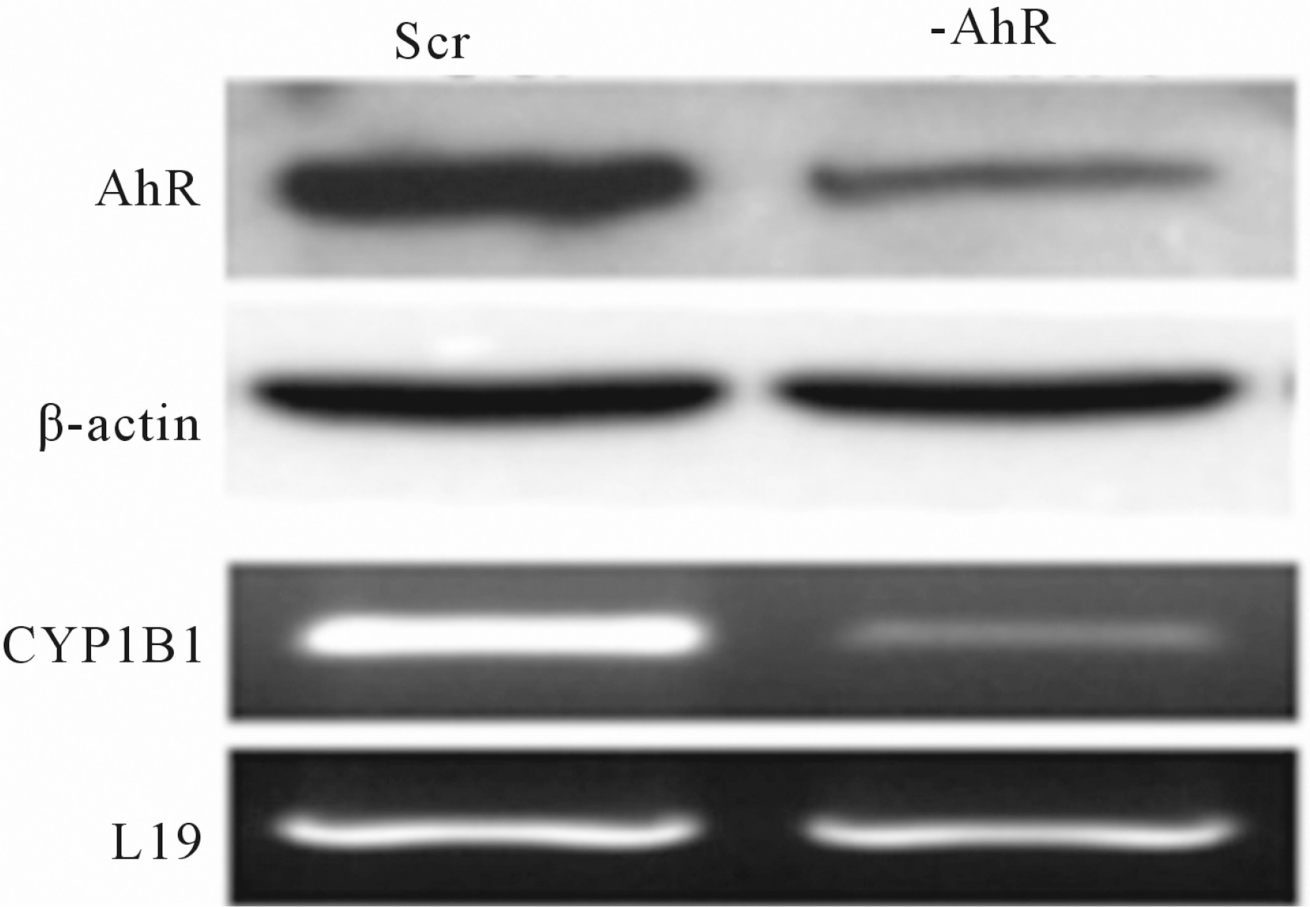
Prostate cancer cells transfected with a scrambled sequence (SCR) have high basal CYP1B1 expression shRNA reduction in AhR protein expression (-AhR) decreases CYP1B1 Mrna levels (from Tran *et al*., 2013, with permission).

Our findings and others clearly affirm the notion of AhR functioning in the progression of breast and other types of cancers, independent of its known ligands. Structurally, AhR contains both nuclear localization signal and nuclear export signal that are required for nuclear-cytoplasmic shuttling of AhR. Nucleocytoplasmic shuttling of AhR is required for inducible expression of CYP1A1 [26]. AhR responsive gene CYP1B1 is expressed in non-small cell lung cancer cells as well as prostate cancer cell lines in the absence of an exogenous ligand. In both cases, CYP1B1 expression was accompanied by increased AhR expression and constitutive activity of the receptor [27,28] (Figure 3). Depletion of AhR protein resulted in subsequent decrease in CYP1B1 expression, confirming that the basal CYP1B1 expression is regulated by constitutive AhR signaling. In addition, pre-malignant and malignant mammary tissues are reported to constitutively express CYP1 B1 mRNA. In these human and rodent mammary tumors, AhR was also over-expressed and constitutively active [23]. In addition, transient overexpression of AhR into an AhR null cell line also induced ligand independent transcriptional activity [28,29].

The studies of the constitutive AhR signaling showed the differential CYP1B1 and not CYP1A1 expression is regulated by constitutively active AhR. The level of CY P1B1 and not CYP1A1 is more closely associated with AhR overexpression and constitutive activity. In the absence of exogenous ligands, AhR overexpression upregulated the expression of CYP1B1 in the early stage of lung adenocarcinoma [30]. However, suspension of wild-type Hepa-1 cells results in nuclear localization and activation of AhR to enhance expression of CYP1A1 [31]. In addition, loss of cell to cell contact experienced at low cell density also induces AhR transcriptional activity. CY P1B1 reporter activity in cells with loss of contact at low density was comparable to the level of activity produced following TCDD exposure. This process, however, was attenuated by depletion of intracellular calcium [32], pointing to the calcium role in this process.

**Figure 4:**
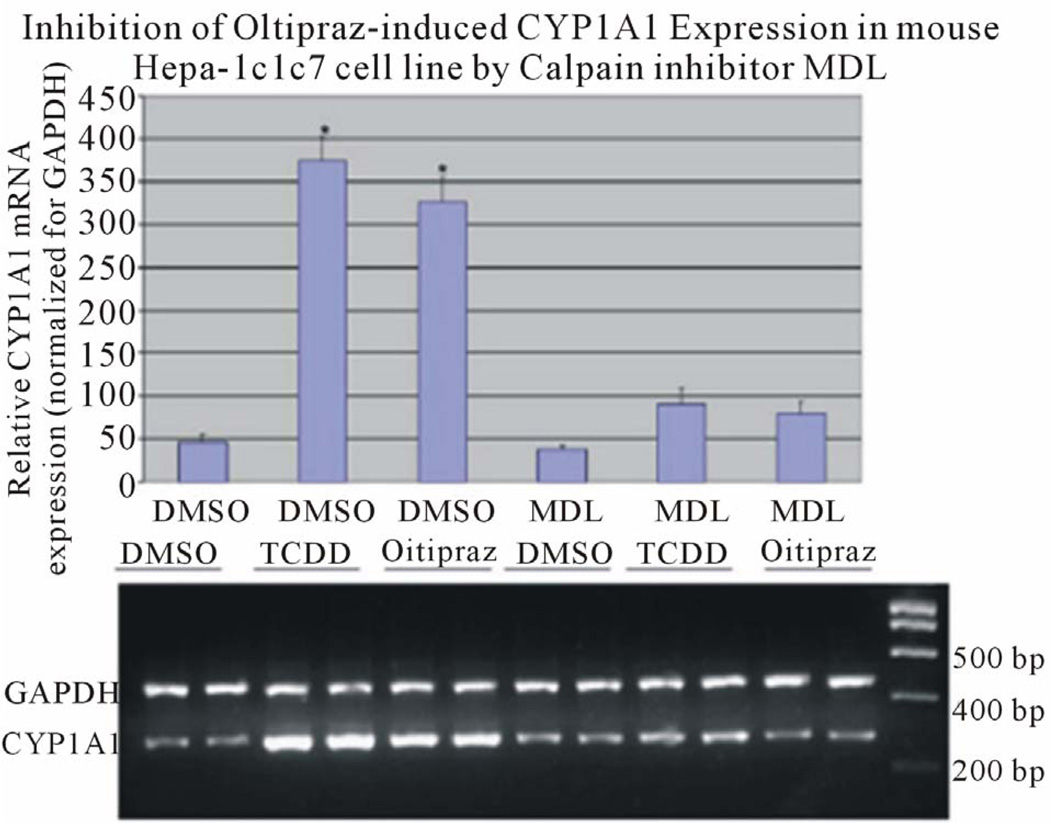
Oltipraz induced CYP1A1 expression to a level comparable to TCDD induction. Oltipraz-induced CYP1A1 expression is inhibited by calpain inhibitor, MDL 28170, in Hepa-1, a murine hepatoma cell line (from Dale & Eltom, 2006, with permission).

We reported previously that activation of calcium-dependent calpain was necessary to mediate ligand-induced activation of AhR. Calpain is a member of a family of cytosolic calcium dependent cysteine proteases known to be involved in a number of cellular processes. Regulation of AhR transcriptional activity has been shown to require calcium changes induced by ligand activation. Results indicate that nuclear accumulation of AhR is dependent on calpain activity. Inhibition of calpain activity with a specific inhibitors blocks transcriptional activity of AhR [33]. Furthermore, chemopreventive agent oltipraz which increases intracellular calcium, induces expression of AhR responsive gene, CYP1A1. Data presented demonstrated that oltipraz induces AhR nuclear translocation through activation of calpain [34] (Figure 4). Additional reports confirmed the enhanced elevation of intracellular calcium in response to AhR activation is associated with enhanced activity of the Ca^2+^/calmodulin (CAM)-dependent protein kinase (CaMK) pathway. In this set of studies, CaMK1α inhibition or knockdown inhibited TCDD induced nuclear translocation of AhR [35].

Although any of these processes could account for the AhR constitutive activation, an endogenous ligand has long been sought after, and many candidates have been reported. The indole metabolite, indoxyl 3-sulfate (I3S) has been identified as a potent endogenous ligand that activates AhR at nanomolar concentrations in primary human hepatocytes [36]. Kynurenic acid, a tryptophan metabolite of the indoleamine-2,3-dioxygenase pathway has also been identified as an endogenous AhR ligand [37]. Competition binding assays revealed these metabolites as true ligands for AhR but the physiological relevance of their expression and activity in breast cancer needs further investigation. Increased understanding of the role of these potential endogenous ligands in breast cancer cells could provide additional insights on the role of AhR in mediating breast cancer progression.

### 2.4. The Cross Talk between AhR and Estrogen Receptor (ER)

Several studies indicate that constitutive AhR signaling is required for crosstalk with ER. Estrogen-induced activation of BRCA-1 transcription requires unliganded AhR binding to the BRCA-1 promoter. It has also been reported that a physical interaction between the hormone-activated ER and constitutively active AhR cooperate to induce estrogen regulated genes [37], whereas activation of AhR by TCDD ablates estrogen induced BRCA-1 transcription [38]. In line of these findings, benzo [a] pyrene (B[a]P), an AhR ligand, also inhibited BRCA-1 expression in MCF-7 breast cancer cells and reduced these cell proliferation in a time and dose dependent manner [39]. A constitutively active mutant of AhR that was designed to mimic continuous TCDD activation also inhibited expression of estrogen-dependent cathepsin D and attenuated the estrogen-induced growth of MCF-7 human breast cancer cells [40]. Collectively, these studies indicate that constitutive activation of AhR is estrogenic while ligand activation of AhR appears to have antiestrogenic effects.

Several reports documented the crosstalk between the ER and AhR ligand-induced signaling pathways. Suppression subtractive hybridization studies using MCF-7 cells identified 33 genes that are induced by estrogen and inhibited by AhR agonist [41]. The AhR agonist-induced decline in estrogen-induced gene expression is a result of AhR inhibition of ER binding to ERE and targeting of ER to proteasome degradation [42]. Mutant cell lines deficient in AhR do not retain the ability to inhibit estrogen induced gene expression upon TCDD exposure [43]. Circulating levels of estrogen are significantly decreased by exposure to TCDD in pregnant rats [44], due to induction of CYP1 B1 and CYP1A1 which metabolizes estrogens [45,46].

### 2.5. AhR Antagonists/Agonists Protect against Tumor Progression

Antagonists for the AhR appear to have protective effects against carcinogen-mediated tumor initiation. Epigallocatechin-3 gallate (EGCG), a green tea polyphenol, reversed epithelial to mesenchymal transition (EMT) in DMBA treated mammary tumor cells. EGCG also reduced levels of c-Rel and the protein kinase CK2 [47]. Considering AhR is a known inducer of c-Rel and EGCG reduced expression of this target gene by AhR ligand DMBA, EGCG is suggested to inhibit EMT through direct inhibition of AhR signaling.

Genistein, a soy phytoestrogen, reduced the overall rate of DMBA induced mammary tumors. The treated rats appeared with larger mammary glands and increased terminal ducts as a result of increased proliferation [48]. Furthermore, genistein was able to inhibit estrogenic effects and induction of cell proliferation by ginsenoside Rg1 in MCF-7 cells [49]. A separate screening for potential AhR antagonist revealed significant suppression of dioxin induced AhR activation by genistein [50]. The studies were performed using Hepa-1c1c7 cells stably transfected with a secreated alkaline phosphatase (SEAP) gene under the control of the XRE/DRE consensus sequences. The established clones secreated SEAP following stimulation with TCDD in a dose dependent manner. Inhibition of SEAP secretion by genistein, therefore, confirms competitive inhibition of TCDD binding to XREs.

Resveratrol is another natural chemopreventive agent that was identified as an antagonist of the AhR [51]. Stilbene derivatives of resveratrol have been developed with high affinity for the AhR [52]. These are of particular interest due to reports that pterostilbene, a natural stilbene isolated from blueberries, has anticancer properties. Pterostilbene inhibited tumor associated macrophage (TAM) induced-migration and invasion of MCF-7 and MDA-MB-231 breast cancer cells. When the breast cancer cells were co-cultured with TAMs, they expressed increased levels of HIF-1α and NF-κB. Pterostilbene inhibited the TAM induced increase in NF-κB. Pterostilbene also suppressed tumor formation in mice inoculated with TAM co-cultured MDA-MB-231 cells [53]. Considering the established role for AhR in modulating NF-κB signaling, it can be surmised that this is an AhR dependent effect.

Ginkgo biloba extracts are known to have estrogenic activity [54]. A CYP1A1 dependent EROD assay revealed increased activity following incubation of MCF-7 cells with ginkgo biloba. CYP1A1 mRNA levels were also elevated following exposure to ginkgo biloba, indicating activation of the AhR [55], and confirming that ginkgo biloba is a natural agonist for the AhR.

### 2.6. AhR as a Transcription Regulator

AhR represents an attractive druggable target due to its ability to regulate a wide range of physiological processes. As a transcription factor, AhR affects a number of genes besides those involved in xenobiotic metabolism. Evidence shows that the four and a half LIM domain 2 (FHL2), a transcriptional coactivator, interacts directly with AhR in MCF7 and PC3 cells to enhance AhR transcriptional activity. However, FHL2 suppressed AhR activity in T47D and LNCaP cells. It’s worth noting that previous reports have shown constitutive expression of CYP1B1 in MCF-7 and PC3 cells while ligand activation is required in T47D and LNCaP cells. These observations suggest that overexpression of FHL2 is required for ligand-independent signaling of AhR to enhance transcription [56]. AhR can also interact with Sp1 proteins to regulate basal expression of some genes; it appears that both the GC-rich motif that bind Sp1 protein and adjacent dioxin responsive elements (DRE) are required for maximal basal expression of Sp1 regulated genes [57].

Altered mammary gland development resulting from *in utero* exposure to AhR ligands is the result of a direct effect on mammary epithelial cells that includes alterations of cell cycle regulator, cyclin D1. Cyclin D1 levels were also decreased in mammary epithelial cells isolated from mice exposed to TCDD [58]. AhR null cells have decreased expression of Cdc2 and Plk, two kinases important for G2/M cell cycle transition [59]. Mouse hepatoma cells deficient in AhR showed decrease proliferation resulting from a prolonged G1 phase [60]. Multiple A549 clones overexpressing AhR have increased proliferation rates proportional to the amounts of AhR [61]. Increases in TGF-β in AhR null cells appear to be the primary factor that causes low proliferation, thus AhR depletion in fibroblast resulted in increased TGF-β gene expression accompanied by decreased proliferation [62]. These studies collectively reveal estrogen-independent mechanisms by which AhR may regulate cell cycle progression.

AhR regulates expression of the zinc finger transcription factor slug which is critical for the induction of epithelial-mesenchymal transition (EMT). AhR directly binds to XREs in the promoter region to enhance transcription of slug [63]. Activation of AhR also represses T-cadherin expression which contains a XRE in the 5’ untranslated region [64]. Therefore, AhR may affect cell adhesion and migration through enhancement of slug and T-cadherin expression. Depletion of AhR expression in mouse embryonic fibroblast results in cytoskeleton alterations due to downregulation of Vav3 expression. Vav3 is a guanosine diphosphate/guanosine triphosphate exchange factor for Rho/Rac GTPases and provides additional insight into AhR’s regulation of cell shape, adhesion and migration [65]. As with slug and T-cadherin, AhR regulates Vav3 signaling by direct binding to the promoter region.

Matrix metalloproteinases (MMP) are key players in cancer invasion and metastasis and provide a possible mechanism by which AhR may modulate the invasive potential of cancer cells. Activation of AhR induces transcription of MMP-9 in advanced prostate cancer cells [66]. TCDD induces expression of MMP-1 through two AP-1 elements in the promoter of the MMP-1 gene [67]. Physical interaction between the RelA subunit of NF-κB and AhR increases transcription of the c-myc oncogene [68]. Modulation of c-myc expression could allow AhR to regulate neoplastic transformation.

## 3. Closing Summary Statement

There is a growing practice of molecularly targeted therapies in oncology for the treatment of malignancy. We now have remarkable success with agents that enable disease specific treatment with reduced normal tissue toxicity. However, as more and more molecularly targeted agents enter clinical evaluation, problems of more clinical remissions are arising, limiting the utility of a targeted agent within certain patient population. Therefore, identification of novel therapeutic targets is essential to combat breast cancers, especially those lacking estrogen receptor/progesterone receptor and ErbB2 receptor (triple negative breast cancer). Interestingly, many of the triple negative breast cancers cells have increased expression of AhR protein [22].

As increased amount of AhR protein contributes to tumor cell aggressiveness and survival, depletion of AhR reduced orthotopic xenograft tumor growth and metastasis *in vivo*; making it an ideal candidate for targeted therapy. More relevantly, depletion of AhR in metastatic breast cancer cell line potentiates the efficacy of chemotherapeutic agents and ionizing radiation, increasing the percentage of cells undergoing apoptosis in response to both treatment modalities [25]. As challenges to targeted therapies include acquired and primary resistance, targeting AhR could be a possible way to circumvent the emergence of targeted therapy resistance and cancer recurrence.

The development of selective AhR modulator (SAhRM) could prove beneficial in preventing breast cancer progression and/or metastasis. Such SAhRM include 3,3’-Diindolylmethane (DIM), which was shown to inhibit cell proliferation by inducing apoptosis and delaying cell cycle progression [69]. Not only can the AhR be targeted independently, it can be targeted in combination with other cancer treatments, such as chemotherapy or radiation therapy. As better understanding of AhR activity in breast cancer has shed light on some aspects of AhR signaling, identifying downstream targets will constitute even better option for targeted therapy, given the essential role of AhR in maintenance physiological functions. In regards to the role of AhR in breast cancer, large clinical studies as well as further investigations into the molecular mechanism of AhR function are essential. Early detection and increasing the list of therapeutic targets remains essential as the fight against breast cancer continues.
